# Association between Gray-Scale Ultrasound Imaging and Serological Creatine Kinase for Quantifying Exercise-Induced Muscle Damage: An Observational Study

**DOI:** 10.3390/bioengineering11010040

**Published:** 2023-12-29

**Authors:** Jorge Buffet-García, Davinia Vicente-Campos, Mónica López-Redondo, Sandra Sánchez-Jorge, Javier Álvarez-González, Gustavo Plaza-Manzano, Tamara Seijas-Fernández, Juan Antonio Valera-Calero

**Affiliations:** 1 Faculty of Health Sciences, Universidad Francisco de Vitoria, 28223 Madrid, Spain; j.buffet.prof@ufv.es (J.B.-G.); davinia.vicente@ufv.es (D.V.-C.); monica.lopezredondo@ufv.es (M.L.-R.); s.sjorge.prof@ufv.es (S.S.-J.); j.alvarezglez.prof@ufv.es (J.Á.-G.); 2 Department of Radiology, Rehabilitation and Physiotherapy, Faculty of Nursery, Physiotherapy and Podiatry, Complutense University of Madrid, 28040 Madrid, Spain; gusplaza@ucm.es (G.P.-M.); tamaraseijasfernandez@gmail.com (T.S.-F.); 3 Grupo InPhysio, Instituto de Investigación Sanitaria del Hospital Clínico San Carlos (IdISSC), 28040 Madrid, Spain

**Keywords:** biomarkers, muscle damage, quadriceps, soreness, ultrasound imaging

## Abstract

Limited evidence has verified if ultrasound imaging (US) can detect post-exercise muscle damage based on size, shape, and brightness metrics. This study aimed to analyze the correlation between creatine kinase (CK) concentration and (as a biomarker of muscle damage) changes in US gray-scale metrics after an exercise-induced muscle damage protocol. An observational study was conducted at a private university lab located in Madrid. Twenty-five untrained and asymptomatic volunteers were enrolled in this study. Baseline demographic data and body composition metrics were collected. In addition, the rectus femoris US data and CK concentration were assessed at baseline and after inducing muscle damage (24 and 48 h later). After calculating time differences for all the outcomes, the correlation between the changes observed with US and biomarkers was assessed. Significant CK concentration increases were found 24 h (*p* = 0.003) and 48 h (*p* < 0.001) after exercise. However, no significant changes in muscle size, shape, or brightness were found in any location (*p* > 0.05 for all). In addition, no significant associations were found between CK changes and US changes (*p* > 0.05 for all). Gray-scale US is not a sensitive tool for detecting muscle damage, as a protocol of exercise-induced muscle damage confirmed with CK produced no significant gray-scale US changes after 24 or 48 h. In addition, US and CK changes after 24 and 48 h were not associated with each other.

## 1. Introduction

Exercise-induced muscle damage (EIMD) commonly occurs as a physiological response to high-intensity or new physical activities, especially those that include eccentric muscle contractions [1]. This damage can occur with a loss of maximal voluntary contraction force and range of motion, and increased soreness, limb circumference, pressure sensitivity, and muscle stiffness [2].

Recently, Owens et al. [3] summarized the mechanisms involved in the primary and secondary muscle damage associated with high-intensity exercise. The primary muscle damage is caused by mechanical loading during exercise, where sarcomeres lengthen heterogeneously until they are beyond myofilament overlap, causing tension on passive structures and leading to fiber disruption. Excitation–contraction (E-C) coupling failure also contributes to primary muscle damage, with reduced force production observed in the days following eccentric exercise. On the other hand, secondary muscle damage involves inflammatory processes that exacerbate tissue damage. Although there are competing theories for explaining muscle damage phenomena, it is generally acknowledged that the initial event after eccentric contraction disrupts the contractile and non-contractile apparatus, followed by membrane damage and subsequent E-C coupling dysfunction.

Therefore, EIMD results in a cascade of structural and functional alterations within the muscle tissue, which can manifest as temporary muscle soreness, inflammation, weakness, and a decline in muscle strength and performance [2,3,4,5]. Understanding and accurately evaluating EIMD is of paramount importance for researchers and practitioners in the fields of sports medicine, rehabilitation, and exercise science. Such knowledge can inform the design and monitoring of effective training programs, injury prevention strategies, and recovery interventions for athletes and individuals engaged in physical activity [6,7].

Multiple invasive and non-invasive instruments have been proposed to assess exercise-induced muscle damage aspects including mechanical muscle damage (i.e., muscle biopsy, magnetic resonance imaging (MRI), diffusion tensor imaging (MRI/DTI), and electromyography), inflammation (i.e., muscular proteins including creatine kinase (CK) and myoglobin, muscle biopsy, pro- and anti-inflammatory cytokines, C-reactive protein, and lactate dehydrogenase), muscle soreness (i.e., pain and perceived exertion scales), performance (i.e., vertical jump and maximal voluntary contraction force), range of movement, and muscle regeneration (i.e., muscle biopsy, MRI, MRI/DTI, and ultrasound imaging (US)) [2].

Among the serum biomarkers, CK concentration is one of the most widely used and extensively studied indicators of EIMD [8,9,10,11]. Elevated CK levels are indicative of muscle cell membrane disruption and the subsequent leakage of intracellular enzymes into the bloodstream, reflecting the severity of muscle damage [8,9,10]. However, the use of serum biomarkers like CK presents several limitations, such as its invasive nature, inter-individual variability, and the influence of factors unrelated to muscle damage (e.g., exercise intensity, training status, and genetics). Moreover, CK measurements may not provide a comprehensive picture of the extent and dynamics of muscle damage and recovery, as they are limited to the detection of intracellular enzyme release without direct assessment of the affected muscle tissue [11].

In recent years, ultrasound (US) imaging has emerged as a promising non-invasive, real-time, valid, and reliable tool for the assessment of muscle tissue characteristics, including size, shape, and echogenicity (brightness) [12]. In addition, current advances in panoramic US allow the visualization and assessment of large muscles that cannot be assessed with traditional two-dimensional (2D) US, producing no image deformation or brightness alteration [13]. The advantages of using US for EIMD evaluation are manifold. First, US can visualize and quantify structural changes within the muscle, such as muscle fiber disruption, connective tissue alterations, and the presence of intramuscular fluid, which are indicative of muscle damage and inflammation [14]. Second, US allows for the detection of edema and inflammation, which can be associated with the severity of muscle damage and the subsequent healing response [15]. Third, US enables longitudinal and safe monitoring (as non-ionizing radiation is involved) of muscle tissue changes, providing insights into the recovery process over time and informing individualized intervention strategies [16]. Moreover, US imaging can be easily performed at the point of care or in the field, reducing the need for laboratory testing and facilitating the individualized monitoring of athletes and patients [17].

Since US has been used in other circumstances for assessing histological metrics based on US brightness [14,18,19,20], this study aimed (1) to investigate whether gray-scale US metrics can detect EIMD after a specific exercise protocol by analyzing size, shape, and brightness changes 24 and 48 h after the exercise and (2) to analyze if these potential US changes are correlated with CK concentration changes as a gold-standard serological biomarker of muscle inflammation.

## 2. Materials and Methods

### 2.1. Study Design

Between June 2022 and November 2022, a cross-sectional observational diagnostic accuracy study was designed to analyze short-term rectus femoris US (i.e., muscle size, shape, and brightness) and CK concentration changes after an eccentric lower limb exercise protocol for inducing EIMD and the association between the image and serological changes. This data collection was conducted at a private University located in Madrid (Spain). While composing this report, adherence to strengthening the reporting of observational studies in epidemiology (STROBE) guidelines [21] and enhancing the quality and transparency of health research (EQUATOR) guidelines [22] was maintained. The entire protocol was supervised and approved by the Ethics Committee of Francisco de Vitoria University prior to the data collection.

### 2.2. Participants

After posting local announcements around the campus, a sample consisting of asymptomatic sedentary volunteers was recruited via convenience sampling. To be eligible for participation in this study, subjects had to be not involved in any regular physical activity or conditioning program and fill out a standardized clinical history declaring good health status and absence of smoking habits. No strict limits on weight, height, or body mass index were considered to obtain better generalizability of the results. However, age cut-offs were established (25 to 55 years) since middle-aged individuals seem to experience greater symptoms of muscle damage and impaired recovery than young subjects [23]. Participants with cardiorespiratory conditions, musculoskeletal injuries, inflammatory disorders, or any other medical condition not compatible with extenuating exercise performance or altering the normal muscle tone (e.g., consumption of muscle relaxants) or US visualization (i.e., neuromuscular conditions) were excluded from this study. Every participant who fulfilled the eligibility criteria was requested to read and provide their signed informed consent in writing prior to the commencement of data gathering.

### 2.3. Exercise-Induced Muscle Damage Procedure

Before starting the EIMD protocol, all participants were contacted to avoid any form of extraordinary physical activity or exercise 48 h before starting the EIMD procedure. In addition, all participants were asked to avoid any form of exercise or recovery strategies 48 h following the EIMD procedure.

During five weeks of data collection, 5 groups of 5 individuals (1 group a week) were cited three days a week at the same hour (10:00 am). On the first day, baseline characteristics were collected one hour prior to the EIMD protocol, including sociodemographic data, rectus femoris US image acquisition, and CK concentrations. Then, participants were scheduled again after 24 and 48 h to measure CK concentrations and acquire new US images.

Based on the procedure described by Hill et al. [24], all participants were asked to perform 100 drop jumps (jumping maximally upon landing from a 0.6 m platform), divided into 5 sets of 20 jumps each considering a 10 s timeframe between each jump and a 2 min resting period between sets.

### 2.4. Assessments

#### 2.4.1. Demographic and Body Composition Features

A standardized self-reported form was filled out by all participants including information about their age, gender, and height. Body weight (kg), body mass index (BMI = kg/m^2^), skeletal muscle mass (kg), body fat mass (kg), and water volume (L) were acquired using the InBody 770 bioimpedance device (Biospace, Urbandale, IA, USA). An independent laboratory technician conducted these measurements one hour before starting the EIMD protocol, as described by Larsen et al. [25].

#### 2.4.2. Muscle Damage Serological Biomarkers

The procedure followed to obtain the CK concentration was described in a recent study [26]. A blood sample (0.25 mL) was drawn from the second finger capillaries at baseline and after the EIMD protocol (after 24 and 48 h). After the blood extraction, the sample was centrifuged in order to measure the CK concentration with the portable biochemical kit SpotchemTM EZ SP-4430 (Menarini Diagnostics, Winnersh-Wokingham, UK) and soft reagent stripes (Arkray Factory Inc., Shiga, Japan). Since the plasma enzyme activity was obtained in micro-Katal per Liter units (µkat/L), a conversion to the International System of Units (SI) was calculated, considering 1 µkat/L to be 60 International Units per Liter (U/L).

#### 2.4.3. Ultrasound Imaging

Since the measurement of the rectus femoris muscle with panoramic US requires a trained operator [27], and test–retest reliability shows less score variability if a single examiner is involved [28] based on the intra-class correlation coefficients (ICCs) reported in the literature (ICC = 0.963 to 0.991 for intra-examiner reliability, and ICC = 0.946 to 0.986 for inter-examiner reliability) [29], a single examiner with more than 10 years of experience assessing and managing musculoskeletal conditions with US was involved to conduct all the image acquisitions and measurements.

Regarding the materials, a Canon Aplio A and a PLT-1005-BT 14L5 (5–14 MHz) linear transducer (Canon Medical Corp, Tokyo, Japan) were used. The console settings were standardized for all the imaging acquisitions (frequency = 12 MHz, gain = 78 dB, dynamic range = 75 dB, and depth = 4.5 cm).

Participants were placed in a supine position with both legs extended and relaxed with a pillow under their knees. Two scanning levels were set:

- At the middle distance between the origin of the rectus femoris muscle (anterior inferior iliac spine) and the insertion of the quadricipital tendon (upper limit of the patella base), both identified with US and marked with a skin marker pen.
- At the middle distance between the anterior inferior iliac spine and the mid-point marked previously (25% of the thigh length).

**Figure 1:**
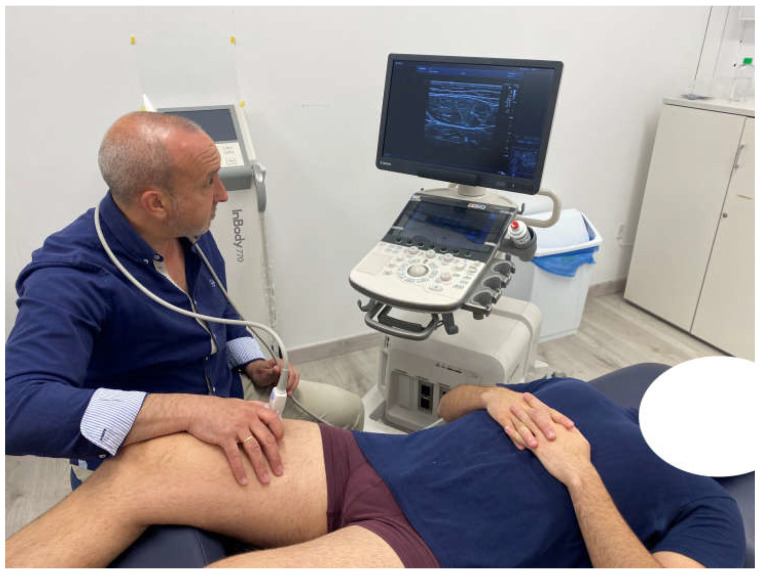
Participant, examiner, and transducer positioning during the image acquisition.

**Figure 2:**
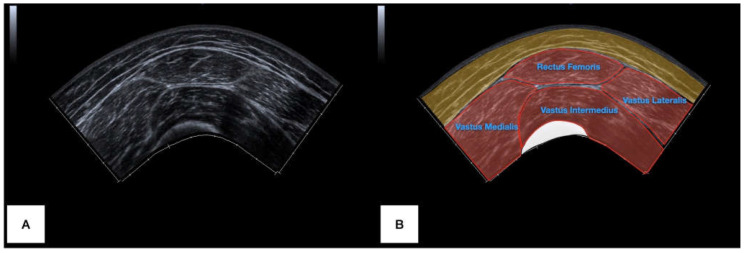
Example of raw panoramic US image acquired (**A**), highlighting the anatomical structures visualized (**B**).

To acquire the panoramic US images, the transducer was placed at the anterolateral aspect of the thigh until locating the sartorius muscle and visualizing it in the lateral extreme of the image. Then, the US computed and generated a real-time panoramic cross-sectional image while laterally gliding the transducer, controlling a constant speed at a rate of approximately 5 cm per second. During this gliding process, the examiner applied the lightest pressure with the transducer as possible. Finally, the image was frozen and saved when the rectus femoris muscle (totally visualized) and the vastus lateralis muscle (partially visualized) were covered. The participant, transducer, and examiner positioning, and the panoramic US image acquired are illustrated in Figure 1 and Figure 2, respectively.

After saving and exporting all images as a DICOM file, the image analyses were run and transformed into 32-bit images (256 gray-scale images) with the ImageJ software v.1.42 (National Institutes of Health, Bethesda, MD, USA) for Mac OS. Using a digital caliper, the RF was carefully contoured, trying not to include the internal fascia of the muscle. Then, the cross-sectional area (the two-dimensional extent of the muscle), mean brightness (the mean brightness of the pixels selected within the muscle area contoured in a 0–255 grayscale), and aspect ratio (calculated as the division between the major axis and the minor axis) were calculated.

### 2.5. Statistical Analysis

Statistical analyses were conducted using the Statistical Package for the Social Sciences (SPSS v.27, Armonk, NY, USA) on Mac OS, with the two-tailed significance threshold set at *p* < 0.05. First, the normal distribution of the data was verified using histograms and Shapiro–Wilk tests (considering a normal distribution as *p* > 0.05). Next, the overall sample data (i.e., sociodemographic and body composition data, CK concentration, and US muscle size, shape, and brightness) were described using descriptive statistics. In addition, sociodemographic and body composition data were reported independently by gender and US characteristics by gender and side, using Student’s *t*-test to analyze between-group differences with a 95% confidence interval.

Next, the effects of the EIMD time on muscle damage, size, shape, and brightness were analyzed by calculating an analysis of covariance (ANCOVA). Baseline values were used as covariates and time (baseline, 24 h later, and 48 h later) as a within-subject factor. Given the multiple outcomes analyzed, a Bonferroni post hoc correction for multiple testing was carried out, such that only *p* < 0.017 (=0.05/3) was considered to be significant [30]. In addition, the effect size was reported by calculating the partial eta squared (η_p_^2^). Cut-off values were reported for small, medium, and large effect sizes (η_p_^2^ = 0.01, 0.06, and 0.14, respectively) [31].

Finally, the correlation between CK and US changes as indicators of EIMD was calculated using Pearson’s correlation coefficients (r). These coefficients were utilized to evaluate both the direction (with positive values indicating a proportional association and negative values indicating an inversely proportional association) and the strength of the associations (where absolute r values from 0.0 to 0.3 were deemed poor, 0.3 to 0.6 as fair, 0.6 to 0.8 as moderate, and 0.8 to 1.0 as strong) [32].

## 3. Results

Out of the 32 individuals who expressed interest in participating in this study, 30 met the inclusion criteria established, and 4 were excluded (n = 1 reported neck pain and n = 3 reported considerable recreational physical activity). Consequently, 28 volunteers were included in the data collection, completing the baseline data collection and the EIMD protocol. However, during the follow-up, three participants were lost. This led to a total of 25 participants completing this study with no CK data loss and acquiring a total of 300 US images (50 US images at the middle third and 50 images at the distal third at baseline, after 24 h, and after 48 h) and AS muscles being examined, obtaining a total of 136 US images.

Table 1 summarizes the sociodemographic and body composition characteristics by total samples and comparing genders. The gender comparison showed males and females to have a comparable age and body fat mass (*p* > 0.05). However, males were significantly taller (*p* < 0.001), heavier (*p* < 0.001), and overweight (*p* = 0.006), and had greater skeletal muscle mass (*p* < 0.001) and water volume (*p* < 0.001).

Participants’ rectus femoris US characteristics are reported in Table 2. Despite the body composition and sociodemographic differences between genders explained previously, no statistically significant muscle area, shape, or brightness differences were found between males and females at baseline (*p* > 0.05 for all). In addition, no side-to-side muscle size, shape, or brightness asymmetries were found in males (*p* > 0.05) or females (*p* > 0.05).

Ultrasound imaging and creatine kinase changes derived from the EIMD protocol are analyzed in Table 3. The ANOVA interaction effect revealed no significant US (muscle size, shape, and brightness) time changes in the proximal (*p* > 0.017 for all) or the middle (*p* > 0.017 for all) third. However, the CK concentration significantly increased 24 h (*p* = 0.003) and 48 h (*p* < 0.001) after the EIMD protocol.

Finally, the associations between the changes observed in the gold-standard inflammatory indicator of muscle damage (CK concentration) and the proposed method (US metrics) are reported in Table 4. The correlation analysis revealed no statistically significant associations between the CK concentration changes and the US metrics assessed in this study either after 24 h or 48 h (*p >* 0.05 for all).

## 4. Discussion

To the authors’ knowledge, this is the first study investigating the association between serological biomarkers of muscle damage and gray-scale US metrics to explore US as a non–invasive tool for assessing EIMD. After analyzing the results, the main findings were an absence of US changes 24 and 48 h after performing the EIMD protocol, despite this damage being confirmed with a serological biomarker. In addition, there was a lack of significant correlations between the CK changes and US metrics. For these reasons, the initial hypothesis was refused, and based on our results, there is no evidence to support the use of US for monitoring EIMD. Therefore, it is not possible to regulate the exercise load using B-mode US as an alternative to CK analysis. However, it should be noted that one potential reason explaining the lack of correlation or US changes after the exercise protocol is the small effect size of the CK increase after the exercise protocol in comparison with other studies [33]. Further research is needed that induces greater muscle damage in order to confirm the absence of correlation or confirm the sensitivity hypothesis proposed.

A recently published review analyzed whether other US technologies, such as shear wave elastography, could be used for EIMD [34]. The literature included in this review indicates that the shear wave speed and Young’s modulus increase after eccentric exercise in the upper and lower limbs, maintaining these changes some days following the exercise performance. However, these findings are still controversial as other studies have found opposite results, such that stiffness changes might depend on exercise characteristics and critical methodological limitations (i.e., elasticity properties depend on the muscle length during the testing procedures [35,36]). In addition, none of the articles included in this review reported the use of biopsy-based measures for confirming the damage magnitude.

Although a direct comparison between US and the gold standard has not been reported in the literature, other indirect measures of muscle damage have been analyzed. Lacourpaille et al. [35] conducted a study analyzing blood tests (CK and aldolase levels), shear wave elastography (shear wave speed), and MRI (short-TI inversion recovery signal hyperintensity and T1 contrast enhancement) in patients with myositis. Their results showed increased CK and aldolase levels and shear wave speed values in this cohort compared with controls, with a significant association between CK levels and shear wave speed. Nevertheless, it was a cross-sectional study analyzing the discriminative capacity between asymptomatic and clinical subjects, and no longitudinal changes were included in the associative analyses.

On the other hand, Andonian et al. [37] tested shear wave speed and blood biomarkers (CK, myoglobin, lactate dehydrogenase, lactate, C-reactive protein, serum uric acid, serum creatinine, glomerular filtration rate, sodium, potassium, serum total protein, plasma osmolality, hematocrit, white blood cells, and neutrophils) in 50 volunteers before, during, and after an extreme mountain ultra-marathon, finding significant correlations between elasticity changes and creatinine, glomerular filtration rate, plasma osmolality, and white blood cell changes but not with any other biomarkers (including CK).

These analyses have been replicated in other circumstances such as trail running events, finding significant CK and shear wave speed increases [38]. However, the lack of association between shear wave elasticity and CK levels seems to be consistent in other studies performing eccentric exercises [39,40].

### Limitations

Although the strengths of this research have been described in this manuscript, this research is not free of limitations. One such limitation is our target restriction to a single muscle. As a result, we cannot be sure that using US in other muscles involved in the EIMD protocol could be more sensitive to detect damage-related changes. Further research is needed that explores other structures under different protocols in order to confirm these preliminary findings. Secondly, even if the sample analyzed in this study had a normal distribution and the characteristic ranges were considerable, the sample size was not free of risk for type II errors. Future research should calculate the minimum sample size needed in order to obtain appropriate statistical power. Finally, the CK changes obtained in this study were not as large as in other previous studies [33], and, consequently, new questions arise (e.g., is the lack of US changes due to a lack of correlation or is it a consequence of not inducing enough muscle inflammation and attributable to a lack of sensitivity?). Even if the initial idea for monitoring the exercise with US is to reduce the risk of EIMD by regulating the load (involving slight CK increases), further research inducing a greater CK increase is needed to solve this controversy.

## 5. Conclusions

This study found that a protocol of EIMD performed by sedentary asymptomatic individuals induced significant CK increases after 24 and 48 h. However, no significant rectus femoris size, shape, or brightness changes were detected with US in this time frame, either in the proximal or the middle third of the thigh. In addition, the correlation analyses revealed that CK changes were not correlated with the cross-sectional area, aspect ratio, or mean echo intensity changes. Therefore, these results indicate that gray-scale US cannot detect EIMD to monitor exercise loads. Further research is needed that applies other exercise protocols and selects other muscles in order to corroborate these findings.

## Figures and Tables

**Table 1:** Participants’ sociodemographic and body composition characteristics.

Variable	Total Samples (n = 25)	Gender		
		Male (n = 11)	Female (n = 14)	Difference
Sociodemographic Characteristics				
Age (y)	36.0 ± 9.7	37.5 ± 11.1	34.8 ± 8.8	2.6 (−6.4;11.7); *p* = 0.545
Height (m)	1.68 ± 0.09	1.76 ± 0.04	1.62 ± 0.07	0.13 (0.08; 0.18); *p* < 0.001
Weight (kg)	71.3 ± 19.4	85.2 ± 13.7	60.3 ± 16.0	24.9 (12.3; 37.4); *p* < 0.001
Body mass index (kg/m^2^)	24.7 ± 4.5	27.3 ± 3.4	22.6 ± 4.2	4.8 (1.5; 8.0); *p* = 0.006
Body Composition Characteristics				
Body fat mass (kg)	21.2 ± 8.8	23.7 ± 7.9	19.3 ± 9.3	4.4 (-2.8;11.7); *p* = 0.219
Skeletal muscle mass (kg)	27.9 ± 7.9	35.0 ± 5.0	22.4 ± 4.6	12.6 (8.6; 16.6); *p* < 0.001
Water volume (kg)	36.7 ± 9.5	45.1 ± 5.9	30.1 ± 5.7	15.0 (10.2; 19.8); *p* < 0.001

**Table 2:** Participants’ rectus femoris US baseline characteristics.

Variable	Gender			Side		
	Male	Female	Difference	Right Side	Left Side	Difference
Proximal third (n = 50)						
Area (cm^2^)	12.1 ± 2.8	11.3 ± 2.6	0.8 (−0.8;2.3); *p* = 0.317	12.0 ± 2.8	11.2 ± 2.6	0.8 (−0.7;2.3); *p* = 0.292
Mean echo intensity (0–255)	50.5 ± 8.3	49.3 ± 11.7	1.2 (−4.8;7.1); *p* = 0.692	50.4 ± 9.0	49.3 ± 11.6	1.0 (−4.9;6.9); *p* = 0.732
Aspect ratio	2.8 ± 0.3	2.5 ± 0.5	0.3 (0.1;0.5); *p* = 0.016	2.6 ± 0.5	2.7 ± 0.4	0.1 (−0.1;0.4); *p* = 0.342
Middle third (n = 50)						
Area (cm^2^)	14.8 ± 8.1	14.4 ± 2.9	0.5 (−2.8;3.8); *p* = 0.784	15.3 ± 2.7	13.8 ± 7.6	1.5 (−1.8;4.7); *p* = 0.369
Mean echo intensity (0–255)	43.2 ± 9.9	40.9 ± 11.5	2.3 (−3.9;8.5); *p* = 0.463	41.5 ± 10.2	42.3 ± 11.5	0.9 (−5.3;7.0); *p* = 0.777
Aspect ratio	2.6 ± 0.2	2.6 ± 0.5	0.0 (−0.2;0.3); *p* = 0.819	2.6 ± 0.4	2.6 ± 0.4	0.0 (−0.2;0.2); *p* = 0.815

**Table 3:** Ultrasound imaging and creatine kinase time change analysis.

		Measurement	Score	ANOVA Interaction Effect	Time Difference	
					24 h	48 h
Ultrasound imaging—rectus femoris characteristics						
Proximal third	Area (cm^2^)	Baseline (n = 50)	11.6 ± 2.7	F = 1.006 η^2^_p *=*_ 0.014 *p =* 0.368	−0.8 (−2.4;0.8); *p =* 0.702	−0.8 (−2.4;0.8); *p =* 0.637
		After 24 h (n = 50)	10.8 ± 3.3			
		After 48 h (n = 50)	10.8 ± 3.8			
	Mean echo intensity (0–255)	Baseline (n = 50)	49.8 ± 10.3	F = 0.299 η^2^*_p =_* 0.004 *p =* 0.742	−1.3 (−6.6;3.9); *p =* 1.000	−1.5 (−6.8;3.7); *p =* 1.000
		After 24 h (n = 50)	48.5 ± 9.7			
		After 48 h (n = 50)	48.3 ± 12.1			
	Aspect ratio	Baseline (n = 50)	2.6 ± 0.4	F = 1.509 η^2^*_p =_* 0.021 *p =* 0.225	−0.2 (−0.4;0.1); *p =* 0.266	−0.1 (−0.3;0.1); *p =* 0.838
		After 24 h (n = 50)	2.5 ± 0.5			
		After 48 h (n = 50)	2.5 ± 0.4			
Middle third	Area (cm^2^)	Baseline (n = 50)	14.6 ± 5.7	F = 1.706 η^2^*_p =_* 0.023 *p =* 0.185	−1.7 (−3.9;0.5); *p =* 0.205	−1.0 (−3.2;1.2); *p =* 0.852
		After 24 h (n = 50)	12.9 ± 3.5			
		After 48 h (n = 50)	13.6 ± 3.9			
	Mean echo intensity (0–255)	Baseline (n = 50)	41.9 ± 10.8	F = 0.096 η^2^_p *=*_ 0.001 *p =* 0.909	0.5 (−4.1;5.1); *p =* 1.000	−0.3 (−4.9;4.3); *p =* 1.000
		After 24 h (n = 50)	42.4 ± 9.3			
		After 48 h (n = 50)	41.6 ± 8.1			
	Aspect ratio	Baseline (n = 50)	2.6 ± 0.4	F = 1.097 η^2^_p *=*_ 0.015 *p =* 0.337	−0.1 (−0.3;0.1); *p* = 0.621	0.0 (−0.2;0.2); *p =* 1.000
		After 24 h (n = 50)	2.5 ± 0.5			
		After 48 h (n = 50)	2.6 ± 0.5			
Serological biomarkers—creatine kinase (n = 25)						
Concentration (U/L)		Baseline (n = 25)	93.8 ± 52.5	F = 11.319 η^2^_p *=*_ 0.239 *p <* 0.001	130.3 (38.2;222.3); *p =* 0.003	171.0 (79.0;263.0); *p <* 0.001
		After 24 h (n = 25)	224.1 ± 136.1			
		After 48 h (n = 25)	264.2 ± 150.1			

**Table 4:** Pearson’s correlation coefficient calculations for reporting the association between creatine kinase changes and US changes.

	Creatine Kinase Change	
	24 h	48 h
1. Proximal third area change	r = −0.087; *p* = 0.554	r = −0.081; *p* = 0.580
2. Proximal third EI change	r = − 0.034; *p* = 0.817	r = − 0.070; *p* = 0.633
3. Proximal third AR change	r = − 0.149; *p* = 0.308	r = − 0.141; *p* = 0.333
4. Middle third area change	r = −0.124; *p* = 0.402	r = −0.197; *p* = 0.171
5. Middle third EI change	r = 0.196; *p* = 0.183	r = 0.113; *p* = 0.435
6. Middle third AR change	r = − 0.248; *p* = 0.089	r = − 0.087; *p* = 0.549

## Data Availability

The data that support the findings of this study are available on request from the corresponding author (J.A.V.-C.).
